# Factors influencing mental health literacy and its relationship with learning weariness in middle school students: a person-centered latent profile analysis

**DOI:** 10.3389/fpsyg.2026.1749928

**Published:** 2026-02-20

**Authors:** Haijuan Zhu, Rui He, Jianjun Zhao, Zhiyong Yu, Kanghui Hou, Guanghua Pan

**Affiliations:** 1 College of Health Science, Shandong University of Traditional Chinese Medicine, Jinan, Shandong, China; 2 Pingdu Zhaoyang Middle School, Qingdao, Shandong, China; 3 Shandong Mental Health Center, Jinan, Shandong, China

**Keywords:** adolescent, latent profile analysis, learning weariness, mental health literacy, middle school students

## Abstract

**Aim:**

To explore the latent profiles of mental health literacy among middle school students and their relationship with learning weariness.

**Methods:**

A total of 1,174 middle school students were selected and assessed using the Mental Health Literacy Scale and the Learning Weariness Scale for Junior School Students. Latent profile analysis was used to classify the students’ mental health literacy. Multinomial logistic regression was employed to explore the impact of demographic variables on the latent profiles, and the Bolck–Croon–Hagenaars (BCH) method was used to examine the relationship between the latent profiles of mental health literacy and learning weariness.

**Results:**

The mental health literacy of middle school students can be categorized into three latent profiles: the high literacy–balanced type (8.18%), the high cognition–low attitude type (61.50%), and the low literacy–developmental type (30.32%). Significant differences were observed among these latent profiles in both the total score and subscale scores of learning weariness (*p* < 0.001). Specifically, the scores showed a statistically significant pattern (*p* < 0.001) as follows: the low literacy–developmental type > the high literacy–balanced type > the high cognition–low attitude type.

**Conclusion:**

Mental health literacy among middle school students is heterogeneous, and its latent profiles are closely associated with learning weariness. These findings provide a scientific basis and practical support for constructing a positive development system for middle school students.

## 1 Introduction

According to a joint report by UNICEF and the World Health Organization, the overall detection rate of mental health problems among children and adolescents worldwide has been estimated to reach 20%, with moderate to severe conditions representing 10% (Yan et al., 2018). This suggests a high prevalence and a tendency toward chronicity, both of which are emerged as major challenges in the field of public health. It is widely acknowledged that students of middle school age are in a critical period of physical and mental development, as well as academic differentiation. Mental health status has been identified as a key factor influencing development. If these issues are not intervened on time during this stage, it can easily lead to academic and social difficulties, trigger psychological disorders such as anxiety and depression, and persist into adulthood (Smith et al., 2025). Mental health literacy (MHL) has been explained as “knowledge and beliefs about mental disorders which aid their recognition, management or prevention” (Jorm et al., 1997). It reflects a set of multidimensional capabilities for coping with mental illnesses and for maintaining and promoting mental health. The enhancement of this ability has been recognized as an essential strategy for improving overall mental health status. The definition of MHL is anchored on four main components: knowledge about the risk factors and determinants of mental disorders; recognition of the symptoms of common mental illnesses; attitudes toward mental illness or individuals with mental disorders and mental health self-help behaviors, as well as help-seeking and helping behaviors.

It is evident that middle school students generally exhibit insufficient levels of MHL, a condition attributable to their relatively underdeveloped psychological maturity (Huang et al., 2021). A substantial body of research has demonstrated that adequate mental health literacy serves not only as a prerequisite for identifying early psychological problems in adolescents (Razmjoo et al., 2025), but also as a key factor in facilitating active help-seeking behaviors (Al Omari et al., 2022; Rahman et al., 2025). Among common psychological and behavioral issues in adolescents, the emergence of risk behaviors such as non-suicidal self-injury is often associated with insufficient awareness of one’s psychological state and difficulties in adopting adaptive emotional coping strategies. Enhancing MHL has been shown to enhance psychological awareness and emotional regulation capacities, thereby facilitating the substitution of self-injurious behaviors with more adaptive coping strategies and preventing emotional escalation. This may serve as an effective protective factor in reducing the occurrence of such behaviors (Baldini et al., 2025; Günaydin, 2024). Therefore, enhancing MHL is not only crucial for the early identification and intervention of psychological risks but also provides a foundational basis for maintaining positive psychological functioning, accumulating psychological resources, and developing sustainable healthy behavioral patterns among adolescents (Aspeqvist et al., 2025). This is particularly important in the context of learning weariness, where MHL contributes to both the prevention of psychological problems and the promotion of mental well-being.

Meanwhile, with the ongoing advancement of educational reform in China, increasingly stringent demands have been placed on educational quality and the holistic development of students. However, the phenomenon of learning weariness remains prevalent among middle school students. It not only exerts direct effects on their academic performance and cognitive development but is also closely linked to mental health issues such as emotional distress and diminished self-worth. This has emerged as an invisible challenge hindering improvements in educational quality and the healthy development of students (Gao, 2023). At its core, learning weariness is defined as a tendency for students to experience fatigue when their academic lives fail to meet their developmental needs. This phenomenon has been conceptualized as comprising three interconnected dimensions: cognitive, emotional, and behavioral. At the cognitive level, it is characterized by the denial of the meaning and value of learning, which may result in academic burnout. At the emotional level, it is reflected in aversion toward learning, which may further develop into school-related anxiety or fear. At the behavioral level, it involves disengagement from academic activities and is commonly expressed through school refusal or problematic absenteeism (Han et al., 2024). While existing concepts, such as problematic school absenteeism, have been used to describe this phenomenon, they primarily focus on attendance-related behaviors and fail to fully capture its cognitive and emotional dimensions (Hendron and Kearney, 2016). Previous research has demonstrated that mental health education and relevant learning content can enhance psychological resilience and proactive help-seeking abilities among students, thereby improving learning attitudes and alleviating learning weariness. Therefore, the improvement of MHL may serve as a critical entry point for the systematic identification and intervention of learning weariness. This approach holds substantial practical significance for promoting students’ holistic development and fulfilling the fundamental educational mission of fostering virtue.

Furthermore, primary and secondary schools have been identified as optimal settings for mental health promotion and prevention (Amado-Rodríguez et al., 2022). MHL is the comprehensive capacity to recognize and address psychological issues. This capacity can be enhanced through interventions in school settings and is seen as a crucial protective factor against academic stress (Lipson et al., 2022). Recent studies indicate that high levels of MHL are positively associated with greater academic interest and may contribute to alleviating learning weariness (Teixeira et al., 2025). Individuals with high MHL are capable of accurately detecting negative emotions, regulating them actively, seeking help when necessary, coping effectively with academic stress, and potentially lessening the negative impact of psychological distress on learning engagement (Günaydin, 2024; Jung et al., 2017). Conversely, individuals with limited MHL tend to develop maladaptive cognitions and learned helplessness when encountering academic setbacks (Joussemet and Mageau, 2023), which could be associated with academic disengagement and learning weariness. From a cognitive- behavioral perspective, robust MHL enables the mobilization of psychological resources across cognitive, emotional (Chen and Li, 2024), and behavioral domains (Bjørnsen et al., 2017). This may contribute to a foundation for managing academic pressure and sustaining learning engagement (Aller et al., 2024; Conejero et al., 2018), while also offering a viable pathway for substantially mitigating and preventing learning weariness. In this context, MHL is recognized as an adjustable and scalable individual capacity. It functions not only as a key protective factor in addressing learning weariness, but it also needs to be integrated into the broader framework of adolescent mental health promotion systems and public health strategies.

Current empirical research on MHL among middle school students predominantly employs variable-centered approaches, which primarily focus on the measurement and comparison of overall levels. However, within the internal structure of MHL, the knowledge, attitude, and behavioral components do not exhibit synchronous change (Zhai et al., 2025). The traditional variable-centered perspective has limited capacity to capture the heterogeneous structures that exist within the population. Therefore, an individual latent profile structure of MHL-centered latent profile analysis among secondary school students. Based on this approach, differences in levels of learning weariness are examined across distinct MHL profiles, thereby allowing for a more nuanced understanding of the relationship between MHL and learning weariness. This study aims to examine the heterogeneity of MHL among secondary school students and to clarify its association with learning weariness, thereby providing empirical evidence to inform the development of more targeted psychological support and intervention strategies.

## 2 Materials and methods

### 2.1 Participant recruitment and data collection

A cluster random sampling method was employed in this study. Three regular secondary schools in Jinan City, Shandong Province, were selected between June and July 2025. Nine classes per school were randomly selected, resulting in a total of 27 classes across all three schools. All students from these classes were surveyed, yielding a sample of 1,298 middle school students participating in the study. Before data collection, approval for the study protocol was obtained from the author’s institutional review board. Questionnaire administration was conducted only after informed consent was obtained from the homeroom teachers, students, and their parents. The questionnaire did not collect any personally identifiable information, such as names, student IDs, or identification numbers. Participants were informed that withdrawal from the survey was possible at any time.

The inclusion criteria were: (1) aged 10–15 years and currently enrolled as a middle school student, and (2) voluntary participation with informed consent. Students with severe psychiatric disorders or physical diseases were excluded. To ensure data quality, outlier detection and invalid response screening procedures were implemented, resulting in 1,174 valid questionnaires and an effective response rate of 90.45%.

### 2.2 Demographic questionnaire

The development of a demographic questionnaire was undertaken in accordance with the study’s objectives and in consideration of pertinent literature. The data collection process involved the gathering of fundamental demographic information, including gender, household registration, and family economic status. In addition, parental details such as occupation and educational attainment were documented. The collection also encompassed school-related experiences, such as serving as a class leader. Family structure was categorized into two types: intact families (both parents present) and non-intact families (including single-parent, reconstituted, and other family forms).

### 2.3 Mental health literacy scale

The assessment of mental health literacy was conducted utilising the Adolescent Mental Health Literacy Assessment Questionnaire developed by Li et al. (2021). The scale comprises 22 items across four dimensions: Knowledge (6 items), assessing basic mental health knowledge and risk factors; Recognition (5 items), evaluating the ability to identify symptoms of various psychological disorders; Attitude (6 items), measuring stereotypes and stigmatizing attitudes toward mental illness and affected individuals; and Behavior (5 items), assessing self-help and helping behaviors. The scale contains six reverse-scored items. The scores of all 22 items are summed, with a higher total score indicating a higher level of mental health literacy. In this study, the scale demonstrated adequate internal consistency, with a Cronbach’s *α* coefficient of 0.80.

### 2.4 Learning Weariness Scale for Junior High School Students

Learning weariness was measured using the Learning Weariness Scale for Junior High School Students developed by Zhao (2019). This scale consists of 17 items and conceptualizes learning weariness into three core dimensions: (1) Learning Weariness Cognitions (7 items), reflecting cognitive-level rejection of learning activities and learning environments; (2) Learning Weariness Behaviors (6 items), capturing behavioral manifestations of disengagement from learning, including skipping classes and delaying homework; (3) Learning Weariness Emotions (4 items), referring to emotional manifestations characterized by strong feelings of weariness toward learning activities. It employs a 5-point Likert scale (1 = strongly disagree, 5 = strongly agree). The total score is calculated by summing responses across all items. The scale contains no reverse-scored items, with higher scores indicating more pronounced learning weariness. In the present study, the scale showed excellent internal consistency, with a Cronbach’s *α* of 0.96.

## 3 Data analysis

### 3.1 Statistical methods

The present study utilised SPSS 28.0 and Mplus 8.7 to conduct latent profile analysis and regression analysis. Latent profile analysis was performed using item scores from the four dimensions of MHL as manifest variables to identify distinct subtypes reflecting differences in MHL among middle school students. Subsequently, an unordered multinomial logistic regression analysis was conducted using category assignment as the dependent variable, with statistically significant demographic variables from univariate analysis (e.g., gender, grade level) as independent variables. Finally, differences across latent categories of MHL in various dimensions of learning weariness were compared using the Bolck–Croon–Hagenaars (BCH) method. Building upon latent profile analysis (LPA), the influence of this classification on learning weariness among middle school students was further examined using regression analysis.

### 3.2 Common method bias assessment

Additionally, Harman’s single-factor test was conducted to assess common method bias. The results of the factor analysis revealed six factors with eigenvalues greater than 1, with the first factor accounting for 28.86% of the variance. This is below the critical threshold of 40%, indicating that common method bias was not a substantial concern in this study.

## 4 Results

### 4.1 Latent profile analysis of mental health literacy in middle school students

The latent profiles of MHL among middle school students were identified using the scores from the 22 items of the MHL scale as indicators. Models with gradually increasing numbers of latent profiles were subsequently fitted and compared.

A series of models with increasing numbers of latent profiles was estimated and compared. The evaluation of model fit was conducted using a range of criteria, including the Akaike Information Criterion (AIC), the Bayesian Information Criterion (BIC), the sample-size adjusted BIC (aBIC), Entropy, the Lo–Mendell–Rubin test (LMR), and the Bootstrap Likelihood Ratio Test (BLRT). Lower values of AIC, BIC, and aBIC indicate better fit, while higher Entropy values suggest clearer classification. The model fit indices are presented in Table 1. As the number of profiles increased, the AIC, BIC, and aBIC values underwent a consistent decrease. The LMR test proved to be significant for the 2-, 3-, and 4-profile solutions. The BLRT proved to be a pivotal element in the 2-, 3-, 4-, and 5-profile solutions. However, the distinction between the profiles identified in the 4- and 5-profile models is not sufficiently clear, and the classification holds little practical significance. Furthermore, the 5-profile solution contained a group comprising less than 5% of the sample. For the 3-profile solution, the Entropy value exceeded 0.9, which was higher than that of the 2- and 4-profile models. In addition, both the LMR and BLRT tests for this model were statistically significant (*p* < 0.05). Further analysis revealed that in the 3-profile model, the average posterior probabilities for each profile were 0.94, 0.97, and 0.98. This indicates that the 3-profile classification model demonstrates high reliability. Therefore, based on model fit indices, classification quality, and parsimony, the 3-profile model was selected as the final model (Table 1).

**Table 1:** Mental health literacy latent profile model fit indices.

Model	AIC	BIC	ABIC	LMRT	BLRT	Entropy	Class proportions
1	76263.36	76486.36	76346.6				
2	73690.35	74029.92	73817.1	<0.001	<0.001	0.856	30.32/69.68
3	71598.66	72054.79	71768.92	<0.001	<0.001	0.914	30.32/61.50/8.18
4	70636.9	71209.61	70850.68	<0.001	<0.001	0.88	29.05/44.21/18.83/7.93
5	69703.08	70392.35	69960.37	0.048	<0.001	0.888	2.3/35.18/18.48/36.88/7.16

**Figure 1:**
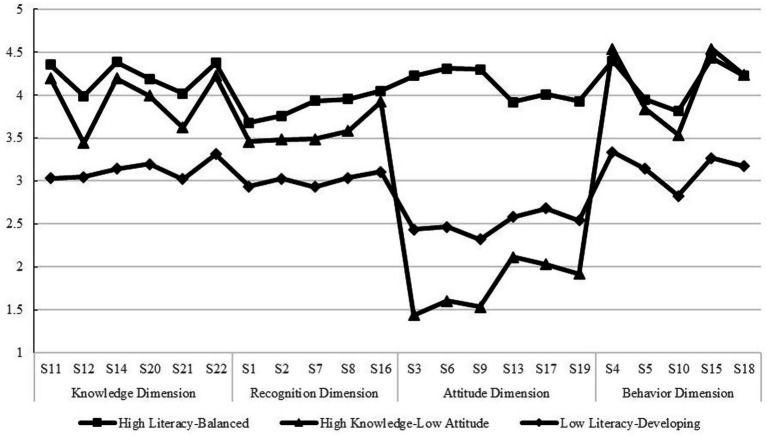
Latent profile analysis of mental health literacy.

As demonstrated in the figure, substantial disparities were identified across the three latent profiles with regard to their ratings on the dimensions of MHL. Profile 1 (C1), comprising 96 students (8.18%), exhibited responses exceeding the average on most questionnaire items. High scores were achieved across all dimensions of mental health literacy (MHL): knowledge, recognition, behavior, and attitude. This indicates that they possess substantial mental health knowledge, strong symptom recognition abilities, and positive attitudes toward mental health issues. They are also capable of both self-help and seeking external assistance. Consequently, this profile was designated as the “high literacy–balanced type”. Profile 2 (C2), comprising 722 students (61.50%), demonstrated comparatively elevated scores on items on the Knowledge, Recognition, and Behavior dimensions. However, these students exhibited notably diminished scores on the Attitude dimension. Their scores represented the lowest among the three identified groups. This suggests that this subgroup of middle school students exhibits a higher level of stigma toward mental illness. It also indicates lower intentions and perceived efficacy in seeking help for psychological disorders. These students demonstrated a satisfactory comprehension of fundamental mental health knowledge. Symptoms of various psychological disorders could also be identified by them with relative accuracy. Their behavioral scores were also relatively favorable. Nevertheless, strong stereotypes and stigmatizing attitudes toward mental illness and affected individuals persisted, revealing a notable internal contradiction characterized by a “cognition-attitude” divergence. This profile was consequently designated the “high cognition–low attitude type”. Profile 3 (C3), comprising 356 students (30.32%), students of this profile scored significantly lower on the knowledge, recognition, and behavior dimensions than the other two types. Although a higher attitude score was observed compared to Type 2, a relatively negative attitude toward addressing mental health issues persisted. This score remained substantially lower than that of Type 1. Consequently, they are considered insufficiently equipped to prevent, identify, or respond to changes in individual mental health and psychological disorders. They exhibited comparatively diminished scores across all dimensions, signifying deficiencies in all domains of MLH and considerable scope for enhancement. Consequently, this group was identified as the “low literacy–developmental type” (Figure 1).

### 4.2 Univariate analysis of latent profiles in middle school students’ mental health literacy

Univariate analysis revealed statistically significant differences among the three latent profiles in terms of family residence location, holding class leader positions, parental education level, and family economic status (all *p* < 0.05), as detailed in Table 2.

**Table 2:** Distribution of mental health literacy latent profiles by sociodemographic.

Item	Profile	High literacy-balanced (*n* = 96)	High knowledge-low attitude (*n* = 722)	Low literacy-developing (*n* = 356)	Test statistic	*p*-value
Gender	Male	41 (42.7)	335 (46.4)	182 (51.1)	3.11	>0.05
	Female	55 (57.3)	387 (53.6)	174 (48.9)		
Only child	Yes	30 (31.3)	267 (37)	127 (35.7)	1.25	>0.05
	No	66 (68.8)	455 (63)	229 (64.3)		
Family residence	Urban	85 (88.5)	691 (95.7)	295 (82.9)	29.8	<0.01
	Rural	11 (11.5)	31 (4.3)	61 (17.1)		
Class leadership role	Yes	48 (50)	294 (40.7)	126 (35.4)	7.3	<0.05
	No	48 (50)	428 (59.3)	230 (64.6)		
Father’s Educational Level	Junior high school or below	26 (27.1)	144 (19.9)	90 (25.3)	14.17^①^	<0.01
	High school	25 (26)	214 (29.6)	122 (34.3)		
	Associate degree	20 (20.8)	126 (17.5)	69 (19.4)		
	Bachelor’s degree or above	25 (26)	238 (33)	75 (21.1)		
Mother’s educational level	Junior high school or below	31 (32.3)	142 (19.7)	98 (27.5)	18.11^①^	<0.01
	High school	23 (24)	215 (29.8)	108 (30.3)		
	Associate degree	19 (19.8)	131 (18.1)	80 (22.5)		
	Bachelor’s degree or above	23 (24)	234 (32.4)	70 (19.7)		
Family structure	Intact family	89 (92.7)	665 (92.1)	316 (88.8)	3.06^①^	>0.05
	Non-intact family	7 (7.3)	57 (7.9)	40 (11.2)		
Family financial status	Very difficult	1 (1)	3 (0.4)	5 (1.4)	8.38^①^	<0.05
	Somewhat difficult	10 (10.4)	52 (7.2)	49 (13.8)		
	Average	59 (61.5)	494 (68.4)	228 (64.2)		
	Above average	25 (26)	169 (23.4)	66 (18.5)		
	Affluent	1 (1)	4 (0.6)	8 (2.2)		

① Fisher’s exact probability test was employed.

### 4.3 Multinomial logistic regression analysis of mental health literacy profiles among middle school students

The three latent profiles of MHL were used as the dependent variable, and a multinomial logistic regression was conducted. Variables identified as statistically significant in univariate analysis were incorporated into the model as independent variables. The association between these demographic variables and MHL was expressed as odds ratios (OR). The latent profile analysis demonstrated excellent classification quality (entropy > 0.90; mean posterior probability > 0.93) with minimal classification error, indicating that the results obtained through the two-step method are highly robust. Furthermore, the coefficients derived from this approach are more easily interpretable and applicable in practice. The reference groups were defined as follows: rural residence, non-class leadership, parental education level (university), and high family economic status. The results of the study are presented in Table 3.

**Table 3:** Logistic regression analysis of sociodemographic variables on mental health literacy latent profiles.

Item	Category	High literacy-balanced vs. high knowledge-low attitude			Low literacy-developing vs. high literacy-balanced			Low literacy-developing vs. high knowledge-low attitude		
		*β (SE)*	OR	CI (95%)	*Β (SE)*	OR	CI (95%)	*β (SE)*	OR	CI (95%)
Family residence	Urban	1.35	0.26^***^	0.133 ~ 0.557	−0.65	1.92^*^	0.946 ~ 3.908	−0.68	0.51^**^	0.299 ~ 0.854
	Rural		1.00	–		1.00	–		1.00	–
Class leadership role	Yes	0.42	1.52^*^	1.084 ~ 2.656	−0.57	0.56	0.355 ~ 0.896	−0.15	0.86	0.654 ~ 1.125
	No		1.00	–		1.00	–		1.00	–
Father’s educational level	Junior high school or below	−0.07	0.93	0.376 ~ 2.112	0.33	1.39	0.561 ~ 3.437	0.23	1.26	0.750 ~ 2.109
	High school	−0.07	0.93	0.458 ~ 1.986	0.43	1.53	0.707 ~ 3.321	0.36	1.43	0.931 ~ 2.185
	Associate degree	0.25	1.28	0.608 ~ 2.575	0.05	1.05	0.492 ~ 2.261	0.30	1.35	0.864 ~ 1.095
	Bachelor’s degree or above		1.00	–		1.00	–		1.00	–
Mother’s educational level	Junior high school or below	0.72	2.05	0.895 ~ 4.972	−0.24	0.8	0.323 ~ 1.979	0.52	1.68^**^	1.000 ~ 2.833
	High school	0.12	1.13	0.530 ~ 2.355	0.15	1.16	0.527 ~ 2.562	0.28	1.32	0.853 ~ 2.039
	Associate degree	0.31	1.37	0.663 ~ 2.875	0.25	1.28	0.589 ~ 2.794	0.58	1.78	1.151 ~ 2.754
	Bachelor’s degree or above		1.00	–		1.00	–		1.00	–
Family financial status	Very difficult	−0.470	0.63	0.025 ~ 15.878	−0.12	0.89	0.042 ~ 18.65	−0.59	0.61	0.088 ~ 4.256
	Somewhat difficult	−0.920	0.40	0.038 ~ 4.124	−0.3	0.73	0.079 ~ 6.890	−1.23	0.32	0.086 ~ 1.219
	Average	−0.960	0.38	0.041 ~ 3.552	−0.77	0.46	0.055 ~ 3.872	−1.74	0.18^**^	0.055 ~ 0.687
	Above average	−0.680	0.51	0.053 ~ 4.821	−1.1	0.33	0.039 ~ 2.868	−1.78	0.17^**^	0.051 ~ 0.672
	Affluent		1.00	–		1.00	–		1.00	–

The group following “vs.” is the reference group in the multiple regression analysis. *p < 0.05, **p < 0.01, ***p < 0.001.

The findings of the study indicated that, in terms of demographic variables, students from urban areas were more likely to be classified into the high literacy–balanced type and the high cognition–low attitude type. It was evident that students who had served as class leaders were more inclined to align with the high literacy–balanced category as opposed to the high cognition–low attitude category.

In relation to family-related factors, students whose mothers had an educational level of junior high school or below were more likely to be classified into the low literacy–developmental type than those with mothers of other educational levels. This is in contrast to the high cognition–low attitude type. Furthermore, in relation to family economic status, students from middle-income or relatively affluent families were more likely to exhibit the high cognition–low attitude type, as opposed to the low literacy–developmental type. However, paternal education level did not demonstrate a significant predictive effect on the latent profiles of mental health literacy.

### 4.4 Relationship between latent mental health literacy profiles and learning weariness in middle school students

To further examine the impact of the aforementioned classification on middle school students’ learning weariness, the BCH method was employed in the analysis. This approach minimizes the adverse effects of initial value differences in latent categories on classification outcomes, allowing the determination of optimal parameter estimates to establish an accurate model that fits the observed data. The research results are shown in Table 4, statistical analysis revealed significant differences among the latent mental health literacy profiles in the total learning weariness score and all its dimensions (*p* < 0.001). Further pairwise comparisons revealed that the low literacy–developmental type scored highest on total learning weariness, learning weariness cognition, and learning weariness behavior, with the high literacy–balanced type ranked intermediate, and the high cognition–low attitude type scoring lowest. Regarding emotional learning weariness, the low literacy–developmental type remained the highest, while the high literacy–balanced type showed no significant difference from the high cognition–low attitude type.

**Table 4:** Differences in learning weariness across mental health literacy latent profiles among middle school students.

Variable	High literacy-balanced (C1)	High knowledge-low attitude (C2)	Low literacy-developing (C3)	BHCχ^2^	LSD *Post Hoc* Test
Total learning weariness score	41.16 (1.87)	37.83 (0.54)	48.29 (0.780)	112.67^***^	C3 > C1 > C2
Learning weariness cognition	13.24 (0.68)	11.74 (0.19)	16.25 (0.29)	153.65^***^	C3 > C1 > C2
Learning weariness behavior	17.34 (0.80)	15.99 (0.24)	20.05 (0.35)	85.95^***^	C3 > C1 > C2
Learning weariness emotion	10.59 (0.50)	10.10 (0.15)	12.00 (0.20)	51.88^***^	C3 > C1 C3 > C2

***p < 0.001.

## 5 Discussion

### 5.1 Latent profiles of mental health literacy and their characteristics

The findings of this study revealed three distinct latent profiles of mental health literacy among middle school students: the “high literacy–balanced type,” the “high cognition–low attitude type,” and the “low literacy–developmental type.” This finding further corroborates the heterogeneous nature of mental health literacy. Among these profiles, the high cognition–low attitude type emerged as the most prevalent, followed by the low literacy–developmental type, whereas the high literacy–balanced type was the least common. This finding is consistent with research by Peng et al., (2025), who also identified comparable profiles among vocational students, thereby supporting the validity of the current classification.

The findings further indicate that approximately 61% of students exhibit an attitudinal lag, characterized by high levels of mental health knowledge and proactive behaviors, yet persistent negative attitudes toward mental health issues. This contradictory pattern suggests that, even when psychological problems are recognized, stigma or shame may impede help-seeking, potentially resulting in secondary harm. Simultaneously, shame is accompanied by an intolerable and persistent sense of inferiority, inadequacy, and worthlessness (Paulo et al., 2019), which can cause secondary harm to both individuals and others concerning psychological problems.

In recent years, multiple studies have confirmed that the knowledge-attitude gap is prevalent among adolescents. Research has revealed inconsistent relationships across various dimensions of MHL (Shi et al., 2019). Huang et al. (2021) found in a survey of Chinese middle school students that self-stigma mediates the relationship between mental health knowledge and the willingness to seek help. This indicates that knowledge education alone is insufficient to effectively alter the negative attitudes of adolescents toward mental health issues. It also confirms previous research suggesting that the assumption of uniformly high or low levels of knowledge, attitudes, and behaviors at the individual level is unreasonable (Sun et al., 2021) and is consistent with the findings of this study. Severe stigma toward mental health issues remains prevalent in society, and such social stigmatization constitutes a significant barrier to adolescents’ engagement in professional psychological services (Maeshima and Parent, 2020). Moreover, negative stereotypes and self-stigma associated with mental illness have been shown to induce resistance to participation in psychological activities (Degnan et al., 2021). Current educational practices, which primarily emphasize the dissemination of mental health knowledge and symptom recognition, fail to facilitate the internalization of such knowledge into positive attitudes, further broadening the cognitive–attitudinal gap. In this study, the labeling of this profile (high cognition–low attitude type) and the interpretation of its cognitive-attitudinal disjunction are based on its response pattern: specifically, high scores across all other dimensional items are observed, coupled with the lowest score on the attitude dimension.

This pattern is consistent with the concept of high social desirability in responses. Yet data from this study cannot directly disclose respondents’ inner motivations or true attitudes. Thus, the label applied remains interpretive, and other explanations may also be possible.

Additionally, approximately one-third of students were classified into the low literacy–developmental type, whereas only 8.2% were categorized as belonging to the high literacy–balanced type. The proportion of students with high-level MHL remains low, a pattern that aligns with existing research (Green et al., 2020) and underscores the need for improvement in adolescents’ MHL. In light of the increasing prevalence of psychological difficulties among adolescents and the widespread concern regarding the mental well-being of middle school students, who are in a critical developmental stage, the comprehensive enhancement of MHL among adolescents is imperative.

### 5.2 Factors influencing mental health literacy

Multinomial logistic regression analysis indicated that family residence, class leadership experience, maternal education level, and family economic status were significant predictors of the latent mental health literacy profiles among middle school students.

Specifically, with respect to family factors, students from urban areas were more likely to be classified into both the high literacy–balanced type and the high cognition–low attitude type. This phenomenon may be attributable to greater access to educational resources and more supportive environments (Huang et al., 2019). Yu et al. (2015) explicitly highlights in their comparative study on adolescents’ MHL that disparities in mental health resources between urban and rural areas persist as a key factor contributing to differences in literacy levels. This study further indicates that such resource inequality not only affects overall MHL levels but may also influence distinct patterns of literacy structure. Urban areas, which benefit from regional economic development, offer easier access to professional psychological knowledge. Moreover, the overall educational environment in urban settings provides external support that facilitates the development of mental health knowledge and recognition skills. In terms of parental education, students whose mothers had lower educational levels were more likely to be categorized as belonging to the low literacy–developmental type, consistent with previous findings (Hunduma et al., 2022). In traditional family structures, mothers typically fulfil the role of primary caregivers and therefore play a pivotal role in shaping the family’s cultural atmosphere (Trujillo and Castañeda-Trujillo, 2024). Lower maternal education may limit access to knowledge and experience, reinforce traditional negative perceptions of mental health issues, foster avoidance-oriented attitudes toward psychological problems, and consequently impede the establishment of a supportive family environment for children’s mental health.

With regard to family economic status, students from more affluent families were more likely to be assigned to the high cognition–low attitude type. Although economic advantages can facilitate access to mental health knowledge, the potential for overprotection and psychological control in affluent families may intensify stigma and negative attitudes toward mental health issues (Chabot et al., 2025). This may consequently contribute to a disconnect between knowledge acquisition and attitude formation. Furthermore, students who served as class leaders were more likely to be included in the comprehensive high literacy–balanced type. This may stem from their increased opportunities to participate in class-level problem-solving, which enhances the application of knowledge through practice. Simultaneously, their heightened sense of responsibility facilitates the formation of positive values (Qi, 2025), thereby promoting the comprehensive development of MHL. This study also enhances understanding of how leadership experiences influence adolescent development, beyond merely their impact on social skills or self-confidence (Griffith and Larson, 2015). It is demonstrated that such capabilities are equally crucial in cultivating mental health maintenance skills, which provide new empirical dimensions to their educational value.

### 5.3 Relationship between mental health literacy and learning weariness

The findings of this study revealed substantial variations in learning weariness scores across the latent mental health literacy profiles, with students in the low literacy–developmental type showing the highest levels of learning weariness.

Research on MHL suggests that students with higher literacy levels are better able to perceive their emotional changes, correctly identify and attribute academic setbacks, and tend to adopt proactive coping strategies (Grasdalsmoen et al., 2020; Li et al., 2025). Such students have been found to mobilize internal and external resources effectively, thereby maintaining self-efficacy and avoiding learned helplessness (Maeshima and Parent, 2020). This process is consistent with psychological resilience development models emphasized in recent years. For instance, it has been shown by research conducted (Lo et al., 2023). That high school students with higher MHL are more likely to proactively seek social support and professional psychological assistance when facing stressful events. This proactive coping behavior is essential for maintaining high self-efficacy under pressure and prevents the depletion of learning motivation. A paucity of learning motivation is prevalent among middle school students, and emotion regulation ability as a fundamental component of MHL plays a key role in their psychological functioning. Students with higher MHL utilize stress management and emotion regulation strategies to mitigate negative experiences from setbacks, thereby preserving motivation and reducing the risk of learning weariness (Ming and Chen, 2020). Correspondingly, students with lower levels of MHL tend to score higher on learning weariness. This pattern likely stems from a relative deficit in the very emotion regulation abilities that constitute a theorized protective mechanism of MHL.

Notably, this study found that students classified as the high cognition–low attitude type had lower learning weariness scores compared with those in the high literacy–balanced type. MHL has been defined as a malleable psychological resource (Habibpour et al., 2025). It has been demonstrated that students who exhibit high cognitive ability but low attitude levels tend to show proficiency in the knowledge and behavioral domains, enabling the application of psychological knowledge as a practical tool when confronting academic pressure. Although their negative attitudes may limit understanding of illness-related concepts (DuPont-Reyes et al., 2020), such attitudes do not necessarily influence academic disposition directly. Conversely, students in the high literacy–balanced type tend to exhibit heightened awareness of psychological states, which may make them more sensitive to subjective stress. When facing academic challenges, they may be more susceptible to ruminative thinking (Sahni and McCabe, 2025), which may result in higher learning weariness scores. Additionally, it is acknowledged that the use of self-report measures in this study may have impacted the results. Students in the high literacy–balanced type, owing to their heightened emotional awareness, may report negative emotions more accurately, whereas those in the high cognition–low attitude type, potentially influenced by stigmatized views toward mental health issues, may underreport their learning weariness, leading to comparatively lower scores.

## 6 Conclusion

In summary, the heterogeneous nature of MHL among middle school students, together with its close association with learning weariness, underscores the necessity for targeted educational strategies. It is imperative that school-based mental health curricula transition from knowledge-dissemination models to integrated approaches that place equal emphasis on attitude cultivation and competency development. Recent intervention studies suggest that contact-based education is more effective than mere knowledge transmission in reducing stigma and improving attitudes (Patten et al., 2012). Therefore, the challenge of “attitude lag” may be addressed in future mental health promotion through experiential activities. Simultaneously, the establishment of screening, classification, and intervention mechanisms is recommended for practical implementation. Differentiated interventions should be implemented based on assessments of students’ MHL types.

Building on this tiered intervention framework, future practices should also consider assessments of students’ family environments and psychosocial resources, while precisely tailoring strategies based on their MHL profiles. Integrating routine MHL assessment into early-stage school psychological screening would allow for the timely identification of students with different needs. For the majority classified as the high cognition–low attitude type, interventions should prioritize destigmatization-focused education, employing experiential methods such as contact-based learning to address the gap between knowledge and attitude. For students of the low literacy–developmental type, more structured and step-by-step comprehensive mental health education courses should be provided, supplemented with necessary individual counseling, to strengthen their foundational MHL. The operation of this support system depends on collaboration among school administrators, mental health teachers, homeroom teachers, and parents. Through policy guidance and resource allocation, sustainable conditions can be created for implementing tiered interventions. Ultimately, this evidence-informed and precision-targeted approach may offer a more tailored and promising pathway to mitigate learning weariness and promote psychological well-being among secondary school students.

### 6.1 Research limitations and future prospects

This study employed latent profile analysis to explore how differences in MHL among middle school students influence their learning weariness. However, several limitations remain: First, the cross-sectional design revealed significant associations between latent categories of MHL and levels of learning weariness, but it cannot establish causality between these variables. Follow-up longitudinal studies should investigate the causes of learning weariness among graduates. Second, common method bias represents a limitation. The data used in this study originated from a single subject and time point, primarily relying on self-reported questionnaire responses. Although evidence suggests that this bias was not severe in this study, measurements of MHL and learning weariness may be skewed by factors such as social desirability bias. Students with negative attitudes toward mental health may underreport their actual distress due to feelings of shame. Future research should therefore integrate diverse data collection methods, such as observation and interviews, while incorporating objective academic indicators to reduce potential errors and enhance data reliability. Finally, limitations exist in the study sample and outcome variables. While the scales demonstrate good reliability and validity in Chinese samples, the cross-cultural generalizability of the conclusions remains constrained and requires validation across different educational systems and cultural contexts. Severe psychological crises stem from complex, multifactorial causes. Subsequent research should incorporate broader mental health assessment outcomes to exert a systematic, strategic, and precision-oriented impact on adolescent mental health prevention efforts.

## Data Availability

The original contributions presented in the study are included in the article/supplementary material, further inquiries can be directed to the corresponding author.
