# Correction: Factors influencing mental health literacy and its relationship with learning weariness in middle school students: a person-centered latent profile analysis

**DOI:** 10.3389/fpsyg.2026.1814544

**Published:** 2026-03-06

**Authors:** Haijuan Zhu, Rui He, Jianjun Zhao, Zhiyong Yu, Kanghui Hou, Guanghua Pan

**Affiliations:** 1 College of Health Science, Shandong University of Traditional Chinese Medicine, Jinan, Shandong, China; 2 Pingdu Zhaoyang Middle School, Qingdao, Shandong, China; 3 Shandong Mental Health Center, Jinan, Shandong, China

**Keywords:** adolescent, latent profile analysis, learning weariness, mental health literacy, middle school students

Authors Haijuan Zhu and Rui He were erroneously omitted as equal contributing authors.

Affiliation “Shandong Mental Health Center, Jinan, Shandong, China” was erroneously given as “Shandong Provincial Mental Health Center, Jinan, Shandong, China.”

The original version of this article has been updated.

